# Perceived Stress: Psychosocial-Sociodemographic Factors as Predictors of Tension, Irritability, and Fatigue Among Ecuadorian University Professors

**DOI:** 10.3390/ijerph22010107

**Published:** 2025-01-15

**Authors:** Henry Cadena-Povea, Marco Hernández-Martínez, Gabriela Bastidas-Amador, Josué Calderón-Muñoz

**Affiliations:** Facultad de Educación Ciencia y Tecnología, Universidad Técnica del Norte, Ibarra 100150, Ecuador; mahernandezm@utn.edu.ec (M.H.-M.); agbastidas@utn.edu.ec (G.B.-A.); jscalderonm@utn.edu.ec (J.C.-M.)

**Keywords:** mental health, occupational disease, stress, teaching

## Abstract

The objective of this study was to identify the factors that best predict variations in tension, irritability, and fatigue (TIF) among university professors in Ecuador. Using a quantitative approach with a non-experimental, cross-sectional design, data were collected from a probabilistic sample of 364 participants. Psychometric measures were adapted and linguistically validated to assess TIF, and participants completed the Perceived Stress Questionnaire, alongside a sociodemographic questionnaire. Written informed consent was obtained, and participation was entirely voluntary. The results indicated that TIF significantly contribute to perceived stress levels among professors. Specific sociodemographic predictors were identified as statistically significant, providing insight into the multifaceted nature of work-related stress in academic settings and its potential implications for health and job satisfaction. The findings underscore the importance of targeted strategies to reduce stress-related outcomes, addressing factors unique to the academic environment in Ecuador. Additionally, while sociodemographic aspects were associated with variations in stress levels, other stress types also triggered TIF among university professors.

## 1. Background

University teaching is inherently multifaceted, requiring a diverse range of competencies, abilities, and skills that are essential for academic management. This includes the effective coordination of educational content to ensure optimal learning outcomes. In addition, the role of university professors extends to interactive management, encompassing effective communication with students, collaboration with administrative staff, and coordination of academic processes. These tasks demand precise time management and adaptability, as professors continuously adjust their teaching practices to meet institutional requirements and respond to diverse student needs.

Consequently, the university work environment is associated with numerous psychosocial risks that can significantly impact professors’ well-being and performance. Studies have shown that academic roles entail high cognitive and emotional demands, often leading to elevated levels of work-related stress [1]. Understanding how these unique challenges in the academic domain contribute to mental health outcomes in professors is essential for addressing and mitigating the negative impacts associated with the profession.

The impact of psychosocial risks on job satisfaction and commitment among university professors has been widely studied. Findings consistently indicate a negative relationship between these risks and job satisfaction [2]. Factors such as excessive work demands, job insecurity, and high workloads have emerged as significant psychosocial risk factors in academic settings, exacerbating stress levels among faculty members [3]. Recent studies highlight the role of job instability and limited institutional support as critical stressors in academia, particularly affecting long-term health and retention rates among professors [4,5,6].

These psychosocial risk factors are inherent to the teaching profession, often leading to chronic work-related stress characterized by both physiological and psychological responses to perceived excessive demands. Such stress can detrimentally impact professors’ physical and mental health, as well as their performance in professional settings. Additionally, organizational culture and the psychosocial safety climate within educational institutions have been identified as significant influences on professors’ well-being. Recent studies have revealed that professors who teach during early morning or late afternoon hours report poorer health perceptions and higher incidences of stress-related symptoms [7].

In addition to institutional factors, external elements also play crucial roles in shaping stress experience for university professors. The COVID-19 pandemic, for instance, has significantly exacerbated existing psychosocial risks, underscoring the urgent need to address these challenges within the educational sector [8,9,10,11]. Furthermore, the work–family interaction has been identified as a key moderating factor in the relationship between work-related stress and emotional exhaustion among university professors [12]. These findings highlight the importance of adopting a comprehensive perspective that considers both organizational and extra-organizational factors to effectively manage the psychosocial risks present in the academic work environment. Prioritizing this holistic approach is essential for supporting professors’ well-being and optimizing their professional performance.

The purpose of this study is to examine the predictive role of psychosocial-sociodemographic factors on levels of tension, irritability, and fatigue (TIF) among Ecuadorian university professors, with perceived stress serving as a central variable. Understanding how these sociodemographic elements contribute to stress-related outcomes is essential, as the academic environment imposes unique demands that can severely impact the mental and physical health of educators. This research aims to provide empirical evidence that can inform the development of targeted intervention strategies, which are crucial for supporting professors’ well-being and enhancing their professional performance. By identifying specific sociodemographic predictors, this study also seeks to add to the growing body of knowledge on occupational stress within educational settings, providing insights that are particularly relevant considering recent global challenges, such as the COVID-19 pandemic, which have intensified the psychosocial risks faced by university professors.

## 2. Materials and Methods

### 2.1. Research Approach

This study employed a quantitative, non-experimental, cross-sectional design with a descriptive and correlational scope, as recommended for studies focusing on identifying associations among variables without manipulating them [13]. The research aimed to analyze how sociodemographic factors predict levels of TIF among Ecuadorian university professors, with perceived stress as the central variable. This approach was chosen to obtain empirical insights into the predictors of work-related stress within the academic sector, particularly focusing on sociodemographic variables as potential sources of variance in stress levels. The descriptive and correlational scope allows for a comprehensive understanding of the relationships between sociodemographic factors and stress dimensions without inferring causality.

### 2.2. Participants

The sample consisted of 364 university professors from the Universidad Técnica del Norte (UTN) in Ibarra, Ecuador. Given the focus on understanding stress predictors across diverse sociodemographic profiles, a probabilistic sampling method was applied to include a wide representation of faculty members across age, gender, ethnicity, and academic departments. This sampling approach was deemed suitable for exploratory studies in which the objective is to capture variability within a particular population [14]. The participants’ ages ranged from 30 to 65 years, with a mean age of 45 (SD = 10). The sample was comprised of 38% female, 61% male, and 1% LGBTI-identified participants.

All participants provided written informed consent after being fully briefed on the study’s objectives, procedures, and confidentiality measures. The study protocol was approved by the Institutional Ethics Committee of ECOTEC, ensuring compliance with ethical standards, including data protection and participant anonymity.

### 2.3. Measurement Instruments

To capture the complexity of perceived stress and its associated psychosocial-sociodemographic factors, two validated instruments were used:

Perceived Stress Questionnaire (PSQ): The PSQ, originally developed by Levenstein and later adapted for Spanish-speaking populations by Sanz-Carrillo et al. [15], was selected for its robust psychometric properties and relevance in assessing general stress levels. The PSQ includes 30 items rated on a 4-point Likert scale, ranging from 1 (“almost never”) to 4 (“almost always”). This scale measures various stress dimensions, including tension, irritability, fatigue, social acceptance of conflicts, energy and enjoyment, work overload, self-realization satisfaction, fear, and anxiety. Previous research supports the PSQ’s reliability and validity across multiple cultural contexts, with Cronbach’s alpha coefficients consistently above 0.80 [16,17,18]. For this study, the PSQ was adapted to reflect the Ecuadorian academic context, achieving a Cronbach’s alpha of 0.85, indicating high internal consistency.

Psychosocial-Sociodemographic Questionnaire: An ad hoc psychosocial-sociodemographic questionnaire was developed based on guidelines from similar studies [19,20]. This instrument gathered detailed information on participants’ gender, age, family relationships, financial responsibilities, educational level, and occupational characteristics. Occupational variables included work experience, employment type (e.g., part-time, full-time, contract, tenure), work schedule (e.g., morning, afternoon, evening), and work conditions. Health-related items covered pre-existing health conditions, dependencies, and lifestyle practices relevant to mental and physical well-being.

### 2.4. Procedure

Data collection was conducted over a one-month period. Initially, the psychosocial-sociodemographic questionnaire was distributed to gather comprehensive demographic, occupational, and health-related information. The PSQ was then administered to assess perceived stress levels and their specific dimensions.

To maximize accessibility and ensure confidentiality, the surveys were distributed through the university’s institutional information system (SIIU), a secure digital platform familiar to all UTN faculty members. Prior to completing the questionnaires, the participants were introduced to the study’s objectives, ethical considerations, and their right to withdraw at any stage without repercussions. An introductory phase in the SIIU system included information on data handling and the confidentiality of responses, followed by the informed consent document, which participants signed electronically.

A preliminary test of both questionnaires was conducted with a pilot group of 150 faculty members to ensure the clarity, reliability, and relevance of the items within the Ecuadorian academic context. Feedback from this preliminary test led to minor adjustments in wording to improve comprehension and contextual alignment. Data from the pilot group were not included in the final analysis, thus maintaining the study’s sample integrity.

### 2.5. Data Analysis

Statistical analyses were conducted using SPSS v. 25. The dataset was organized by merging responses from the sociodemographic questionnaire and the PSQ. Data analysis began with descriptive statistics to outline the participants’ demographic profiles and general stress levels, followed by inferential analyses to identify predictive relationships.

A stepwise multiple regression model was employed to determine the primary PSQ factors associated with higher stress levels among faculty members. This method enabled the identification of the most influential predictors, providing a refined model for understanding sociodemographic determinants of perceived stress. Additionally, an enter-method multiple regression model was developed to predict variations in TIF (tension, irritability, fatigue) based on social acceptance of conflicts, energy and enjoyment, workload, and fear and anxiety.

To further analyze these relationships, linear regression was applied to the model TIF as a function of the predictor variables. This method was selected for its capacity to measure the individual contribution of each predictor to the outcome variable, facilitating a detailed understanding of the factors that most strongly influence stress among professors. The decision to use multiple regression over alternative techniques, such as chi-square analysis, was based on the need to examine linear relationships and the continuous nature of the variables.

Stress levels were categorized as low, medium, or high using the 33rd and 66th percentiles as thresholds. Hypothesis testing employed Pearson’s chi-square test, or Fisher’s exact test when assumptions were not met, with significance levels set at α = 0.05 and α = 0.10, depending on the analysis context. To further ensure statistical power and accuracy, Kendall’s Tau-B correlation coefficient was utilized, which is particularly suited for ordinal data, enhancing the robustness of the findings.

## 3. Results

The PSQ demonstrated robust psychometric properties, with a reliability coefficient of 0.9 for the overall score and 0.87 for the recent score. In this study, the internal consistency of the PSQ was confirmed with a Cronbach’s alpha of 0.792 and a McDonald’s Omega coefficient of 0.833, indicating satisfactory reliability.

The sample of 364 university professors had a mean age of 45 years (SD = 10 years). The majority (90%) self-identified as Mestizo, while the remaining participants identified as Afro descendant (3.6%), Indigenous (0.5%), White (4.1%), and Montubio (1.4%). Among these, 38.2% were exclusively engaged in teaching without research involvement, 44.8% conducted internal research, and 17% were involved in research outside the university. Employment stability varied, with 44.2% of participants holding permanent positions, while 55.8% were on temporary contracts.

Table 1 provides a summary of the descriptive statistics for the six PSQ factors, including minimum and maximum values, as well as the 33rd and 66th percentiles. These percentiles served as thresholds for categorizing stress levels into low, medium, or high categories. Additionally, the distribution of data across these stress levels is presented, providing insights into the stress profile of the sample. The factor with the highest incidence was tension, irritability, and fatigue with an average score of 17.27, which was higher than the other factors in this test.

A multiple linear regression model was developed using the stepwise method to examine perceived stress levels among professors, with predictor variables including TIF, social acceptance of conflicts, energy and enjoyment, workload, self-realization satisfaction, and fear and anxiety. The use of multiple regression was chosen over alternatives like chi-square tests due to the continuous nature of the data and the need to determine the individual contribution of each predictor to the overall stress levels. This approach allows for a more precise understanding of the linear relationships between sociodemographic factors and perceived stress outcomes, aligning with the study’s goal of identifying key predictors of stress. The model yielded a significant result: F(5, 358) = 388.392, *p* = 0.036, with an R² value of 0.844, indicating that approximately 84% of the variance in perceived stress is accounted for by the predictors in the model (see Table 2).

**Figure 1:**
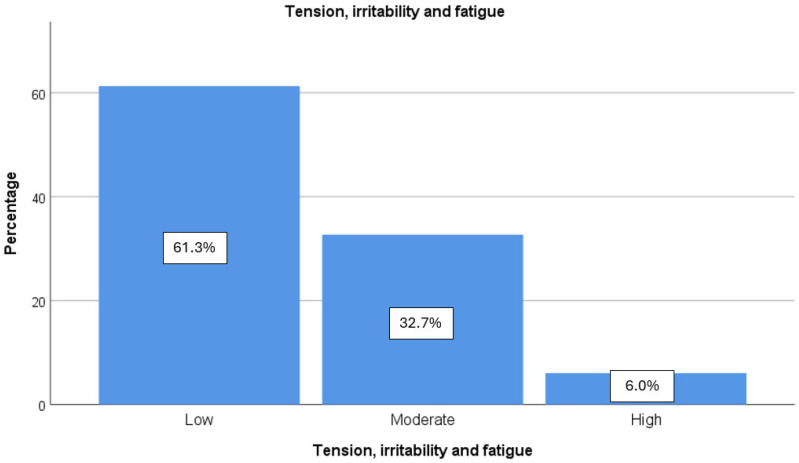
Levels of tension, irritability, and fatigue.

The multiple regression analysis presented in Table 2 indicates that the factor exerting the greatest influence on perceived stress is TIF, warranting further examination of this dimension. As illustrated in Figure 1, approximately 38% of the sample population demonstrates moderate to high levels of TIF, highlighting the prevalence of this stress factor among university professors.

To explore potential causes influencing TIF, this factor was cross-referenced with 54 psychosocial–sociodemographic indicators (Table 3) to identify aspects contributing to the professors’ perceived stress. These findings may inform the design of targeted programs to improve faculty well-being. The results indicate that professors aged 51 and older experience the highest stress levels, followed by those aged 25 to 35. In contrast, professors aged 36 to 50 report comparatively lower stress levels.

Given the impact of both sociodemographic and psychosocial factors on stress and well-being, this table includes variables across both domains. Sociodemographic factors (e.g., age, gender, economic status) and health-related factors (e.g., renal conditions, lifestyle practices) are presented together to provide a comprehensive view of potential influences on perceived stress.

In addition, the findings indicate that professors with neurological conditions experience significantly higher stress levels as compared to their counterparts without such conditions. This suggests that underlying health issues, particularly those impacting the nervous system, may intensify perceptions of stress, which is likely due to the additional physical and mental challenges these conditions impose. Similarly, professors with psychiatric conditions also report elevated stress levels, underscoring the connection between mental health and perceived stress in academic environments. These results highlight the need for targeted mental health support within university settings.

Research engagement emerged as another critical factor in influencing TIF levels. Professors who are not involved in any research activities reported the highest stress levels, followed closely by those conducting internal research within their institutions. In contrast, professors engaged in external research projects reported the lowest stress levels. This pattern suggests that research activities outside the institution may provide a sense of autonomy and fulfillment, potentially reducing stress. The findings point to the importance of fostering research opportunities that offer both an intellectual challenge and a sense of purpose, which could contribute to lowering stress.

The necessity of adapting to new digital tools also correlates strongly with increased TIF levels. Professors who had to integrate digital tools into their teaching or administrative tasks reported significantly higher stress levels than those who did not undergo this adjustment. This association underscores the potential strain involved in adapting to technological changes, especially for those who may lack previous experience or support in digital skill acquisition. These findings suggest that institutions could benefit from providing resources and training to support digital adaptation and alleviate the associated stress.

A similar trend was observed for the shift from in-person to virtual work modalities. Professors who had to develop new competencies to effectively transition to online formats exhibited higher stress levels than those who continued in traditional settings. The increased stress associated with this shift may stem from the need to acquire unfamiliar skills, manage virtual classroom dynamics, and adjust to different pedagogical approaches. This underscores the value of providing structured support and professional development to ease the transition for faculty members required to operate in a virtual environment.

Family dynamics, specifically having children within certain age ranges, appear to influence TIF levels. Professors with children aged 13 to 18 years reported markedly higher stress levels as compared to those with children in other age brackets. Adolescence is a period characterized by developmental changes and challenges that potentially increase parental responsibilities and, consequently, stress levels. These findings suggest that family-related support systems could play a crucial role in reducing stress among professors with teenage children. A multiple regression model was also designed using the intro method to analyze the predictive effect of factors such as fear and anxiety, energy and enjoyment, overload, and social acceptance of conflicts on the TIF of university teachers. Table 4 provides a summary of the model, indicating that approximately 84% of the variation in TIF is explained by the mentioned predictors, with a significance level of <0.001.

The ANOVA results indicate a significant model, F(4, 359) = 470.674, *p* < 0.001, with an $R^{2}$ value of 0.844, suggesting that approximately 84% of the variance in TIF is explained by the predictors. Additionally, the effect size, as indicated by eta squared ($\eta^{2}$), is also 0.84, reinforcing the substantial predictive power of the model (Table 5).

Table 6 presents the coefficients for the multiple regression analysis of TIF with predictors including social acceptance of conflicts, energy and fun, overload, and fear and anxiety. The collinearity statistics (Tolerance and VIF) indicate acceptable levels, suggesting that multicollinearity does not significantly impact the model. The standardized Beta coefficients highlight the relative contribution of each predictor to TIF, with ‘Fear and Anxiety’ showing the highest predictive power (Beta = 0.325, *p* < 0.001).

## 4. Discussion

The findings of this study underscore that specific factors, namely, neurological conditions, psychiatric conditions, challenges in adapting to digital tools, and the need for developing competencies in online education, exhibit significant correlations with levels of Tension, Irritability, and Fatigue (TIF) among university faculty. These results highlight that issues related to mental health and technological adaptation that constitute essential determinants of perceived stress within the academic sector.

This association is corroborated by prior research, which indicates that neurological and psychiatric conditions may intensify stress symptoms, adversely impacting both the physical and mental health of educators [21]. The presence of these conditions suggests that professors contend not only with professional demands but also with personal health constraints, potentially magnifying the effects of TIF.

The study by Redondo-Flores et al. [21] highlights the potential physical symptoms of psychological disorders such as dry mouth, gastritis, and heartburn, which may be associated with increased stress levels among university professors. This suggests that the impact of stress dimensions such as TIF extends beyond psychological well-being to physical health. Xu and Wang [22] explore work-related stress, including research, teaching, and administrative stress, that negatively impacts the life satisfaction levels of university junior faculty members, with emotional burnout as the psychological mechanism responsible. Their study emphasizes the need to address faculty members’ psychological experiences, including the impact of stress dimensions like TIF, on their overall well-being. Meng and Wang [23] provide insights into sources of occupational stress among university professors, highlighting the significant influence of scientific research, professional development, and administrative issues on faculty stress levels. Their study underscores the necessity of addressing university structural limitations and the personal characteristics contributing to occupational stress among faculty members.

The results of this study, which reveal the relationship between psychiatric and neurological conditions and TIF, are consistent with the research by Almhdawi et al. [24], which highlighted the deterioration in the mental and physical well-being of university professors during the COVID-19 pandemic. This connection between mental health and work conditions in a high-demand context underscores the need for interventions that address not only organizational aspects but also psychological support for educators.

Among the predictors, “Fear and Anxiety” emerged as the most influential factor (Beta = 0.325), followed closely by “Social Acceptance of Conflicts” (Beta = 0.314). This finding emphasizes that psychological and interpersonal dynamics are substantial contributors to TIF, suggesting that the emotional demands associated with academic life require careful management. Other significant predictors, including “Workload” (Beta = 0.225) and “Energy and Enjoyment” (Beta = 0.169), underscore the impact of job demands and the necessity for adequate personal coping mechanisms. Together, these factors reflect the complex and multifaceted nature of stress in the academic environment, where professional, interpersonal, and individual challenges interact to shape overall well-being.

The studies by Redondo-Flores et al. [21] also indicate that mental health issues can manifest as physical symptoms, reinforcing the notion that academic stress is a multidimensional condition affecting both the physical and psychological well-being of faculty members.

The difficulties in adapting to digital tools and the need to develop competencies in online education were sociodemographic factors that showed a significant correlation with TIF. These findings align with the study by García-González et al. [25], which observed that the increasing use of information and communication technologies (ICT) in the university context has heightened stress levels among educators due to inadequate skills and insufficient institutional support. During the COVID-19 pandemic, many professors were compelled to adapt rapidly to online teaching, exacerbating their levels of stress and fatigue, as noted in studies such as that by Zheng et al. [26].

Based on the provided references, research specifically focusing on occupational stress among university faculty is limited [19]. Nevertheless, it is evident that the topic of stress among educators, including university professors, has been of interest in various studies [27,28,29,30,31]. These studies have explored different aspects of work-related stress and its impact on educators’ health and well-being. Some studies have also investigated the relationships between stress and specific demographic factors such as age and gender [32,33,34].

According to relevant references, it is evident that the relationship between age and stress levels among professors is a topic of interest in academic research. The study by Teles et al. [16] observed higher levels of emotional exhaustion and depersonalization among older professionals. This finding aligns with the asseveration that professors aged 51 and older experience higher levels of stress. Additionally, Kumari and Hassan’s study [35] compared occupational stress between primary and high school teachers, providing insights into variations in stress levels among different age groups.

Furthermore, Skaalvik and Skaalvik [36]’s research explored teachers’ stress and self-efficacy, indicating that these factors predict engagement, emotional exhaustion, and motivation to leave the teaching profession. This study contributes to understanding how stress levels may vary among different age groups of teachers. Moreover, Jentsch et al. [37]’s study delved into school atmosphere and socio-emotional learning, predicting teachers’ stress, job satisfaction, and teaching efficacy. This research sheds light on factors that may influence stress levels among teachers of different ages.

Conversely, Li and Kou’s study [38] focused on the prevalence and correlates of psychological stress among professors at a key national university in China, which may not directly coincide with specific age groups. Similarly, another study examined the impacts of work-life balance on the emotional exhaustion and well-being of university professors in China, which does not directly address age categories [39].

The relationship between age and stress levels among educators is a complex and multifaceted topic influenced by various factors such as work environment, self-efficacy, and job satisfaction. Findings from Teles et al. [16] and Kumari and Hassan [35] provide valuable information about the differences in stress levels among teachers across different age groups, contributing to a more comprehensive understanding of this issue.

Several studies have addressed how stress factors affect tension, irritability, and fatigue within this specific population. Research such as that of Guerra et al. has highlighted the fact that university teachers may experience occupational stress due to various factors, such as the lack of adequate technology and support in the work environment [40].

Studies such as that of Vilca et al. have explored academic stress in university students, which may be related to the pressure that university teachers face when delivering classes and evaluating students [41]. On the other hand, research such as that of Zheng et al. has addressed the impact of technological stress on teaching staff who have been forced to adapt to online teaching due to the COVID-19 pandemic [26].

The findings of this study carry important implications for health policies and interventions within university settings. First, they suggest a need for targeted mental health support that addresses both individual psychological needs and interpersonal relationship management. Programs designed to enhance conflict resolution skills, reduce anxiety, and foster resilience could prove highly effective in reducing TIF. Furthermore, considering the significant influence of workload on stress levels, universities should prioritize workload management strategies, potentially revisiting faculty responsibilities and providing resources to support a balanced workload.

Institutions may also benefit from implementing professional development programs focused on digital competencies and adaptive resilience, particularly given the technological demands that have emerged in the post-pandemic context. Such programs would not only address immediate skill gaps but also provide faculty with the tools needed to manage future changes in educational technologies. This holistic approach to faculty well-being has the potential to enhance job satisfaction, reduce burnout, and ultimately improve both teaching quality and student outcomes.

## 5. Conclusions

In conclusion, this study underscores the pressing need for universities to develop and implement evidence-based interventions that address the psychological, interpersonal, and professional challenges faced by faculty. By prioritizing mental health support, conflict resolution, workload management, and digital competency training, institutions can create a supportive environment that fosters faculty well-being. Such efforts will not only improve the quality of life for educators but also contribute to the sustainability and effectiveness of higher education institutions globally.

### Limitations and Future Lines

This study, while providing valuable insights into the predictors of TIF among university professors, is not without limitations. First, the cross-sectional design restricts the ability to establish causal relationships between the identified predictors and TIF levels. Future research would benefit from adopting a longitudinal approach to observe the progression and potential causality of these stress factors over time. Longitudinal studies could offer a more comprehensive understanding of how stress evolves and interacts with sociodemographic and psychosocial factors within the academic context.

Second, the sample is limited to university professors in Ecuador, which may impact the generalizability of the findings to other academic settings or cultural contexts. Educational systems, work environments, and cultural expectations vary significantly across countries, potentially influencing the factors that contribute to TIF. Future studies should consider comparative analyses involving diverse academic institutions and regions to determine whether similar predictors hold across different cultural and institutional contexts. Such comparative research could help to tailor interventions that are culturally sensitive and context specific.

This study primarily relies on self-reported data, which can introduce bias, particularly in assessing stress levels and personal health conditions. Self-reported measures may be influenced by social desirability or recall bias, which can affect the accuracy of the data. Future research could incorporate objective measures, such as physiological indicators of stress (e.g., cortisol levels or heart rate variability), alongside self-reported assessments to provide a more holistic understanding of stress experiences among university faculty.

Another limitation lies in this study’s focus on a limited set of sociodemographic and psychosocial factors. While this study highlights the impact of factors such as workload, fear and anxiety, and adaptation to digital tools, other potential variables, such as social support, financial stress, or specific institutional policies, were not included in the analysis. Future research could expand on these variables, incorporating a wider range of predictors that may also contribute to TIF. This expansion would enable a more nuanced model of occupational stress and its influences within the academic setting.

Lastly, the COVID-19 pandemic context, which has profoundly affected higher education, presents a unique limitation in terms of interpreting the results. The shift to online education and the sudden increase in digital demands may have temporarily intensified stress levels, potentially amplifying the significance of predictors like digital adaptation. Future studies should examine whether the importance of these predictors persists as universities stabilize into post-pandemic norms, to determine if these factors are enduring stressors or primarily contextual.

In sum, while this study identifies significant predictors of TIF and suggests relevant interventions, it opens multiple avenues for further exploration. Addressing these limitations will not only refine our understanding of occupational stress in academia but also inform the development of more effective, evidence-based interventions tailored to the unique needs of university faculty worldwide.

## Figures and Tables

**Table 1:** Descriptive statistics of each factor of the CEP.

Statistical Indicators		Tension, Irritability, and Fatigue	Social Acceptance of Conflicts	Energy and Fun	Overload	Satisfaction from Self-Realization	Fear and Anxiety
N	Valid	364	364	364	364	364	364
	Lost	0	0	0	0	0	0
Mean		17.27	11.54	9.59	8.08	4.83	3.71
Median		16.00	10.00	9.00	8.00	4.00	3.00
Mode		14	7	5	8	3	3
Standard Deviation		5.65	4.22	3.70	2.57	1.97	1.44
Minimum		9	7	5	4	3	2
Maximum		36	28	19	16	12	8
Percentiles	25	13.00	8.00	7.00	6.00	3.00	3.00
	33	14.00	9.00	7.00	7.00	3.00	3.00
	50	16.00	10.00	9.00	8.00	4.00	3.00
	66	19.00	13.00	11.00	9.00	5.00	4.00
	75	21.00	14.00	12.00	9.00	6.00	5.00

**Table 2:** Multiple regression model: perceived stress.

Predictor Variables	$F_{\left(5,\;358\right)}$	$R^{2}$	B	${SE}^{\;a}$	p
Model 1	388,392	0.844	−0.028	0.036	0.036
Tension, irritability and fatigue			0.381	0.035	
Social acceptance of conflicts			0.273	0.041	
Energy and fun			0.200	0.027	
Overload			0.080	0.027	
Satisfaction for self-realization			0.082	0.039	

Note: ^a^ Standard error.

**Table 3:** Correlations between TIF and the psychosocial–sociodemographic variables.

Variable	TIF	Variable	TIF	Variable	TIF	Variable	TIF
Tension, irritability, and fatigue	1.000	Neurological conditions	−0.115 *	Internet access	0.040	Family burdens aged 4 to 12	−0.066
Work area	0.044	Psychiatric conditions	0.113 *	Vacation	−0.021	Family burdens aged 13 to 18	0.019
Gender	−0.060	Renal conditions	0.058	Difficulty adapting to digital tools	−0.129 *	Other family burdens associated with disability	−0.064
Age	0.051	Completed education level	−0.042	Hourly workload and greater time demand	0.022	Other adult family burdens	−0.050
Marital status	−0.037	Profession	−0.004	Access and quality of internet service in online education	0.035	Other family burdens—domestic activities	0.007
City of residence	0.077	Ongoing education level	−0.053	Technological equipment for online education	0.035	Pets	−0.023
Ethnicity	0.074	Conducting research	−0.078	Development of competencies in online education	0.120 *	Compliance with financial obligations	0.022
Pregnancy status	−0.081	Type of employment relationship	−0.033	Perceived workload in online education	−0.023	Restructuring obligations with financial entities	−0.003
Type of disability	0.059	Work activity time	−0.017	Family environment composition	−0.007	Physical activity	0.057
Metabolic conditions	−0.047	Institutional role	−0.056	Housing contractual situation	0.005	Sexual activity	−0.020
Hereditary conditions	0.019	Weekly workload	0.009	Family burden	0.057	Tobacco consumption	−0.021
Catastrophic/rare/orphan conditions	−0.041	Work modality	0.005	Family coexistence	0.028	Alcohol consumption	−0.003
Cardiovascular conditions	−0.049	Vaccine use	−0.058	Family burdens under 3 years	0.069	Recreational drug use	−0.013

* Correlation is significant at the 0.05 level (two-tailed).

**Table 4:** Summary of the multiple regression model for TIF.

Model	*R*	$R^{2}$	$\mathrm{Adjusted}\;R^{2}$	Standard Error of the Estimate	Change Statistics				
					Change in $R^{2}$	Change in F	gl1	gl2	Sig. Change in F
1	0.916 ^a^	0.840	0.838	2.273	0.840	470.674	4	359	0.000

^a^ Predictors: (Constant), Fear and anxiety, Energy and fun, Overload, Social acceptance of conflicts.

**Table 5:** ANOVA results for the multiple regression of TIF.

Model		Sum of Squares	df	Mean Square	F	Sig.	$\eta^{2}$	$R^{2}$
1	Regression	9724.264	4	2431.066	470.674	0.000 ^a^	0.84	0.844
	Residual	1854.263	359	5.165				
	Total	11,578.527	363					

^a^ Predictors: (Constant), Fear and anxiety, Energy and fun, Overload, Social acceptance of conflicts.

**Table 6:** Multiple regression of TIF.

Model		^a^ Unstandardized Coefficients		^a^ Standardized Coefficients	t	Sig.	Collinearity Statistics	
		B	Std. Error	Beta			Tolerance	VIF
1	(Constant)	1.234	0.412		2.991	0.003		
	Social acceptance of conflicts	0.419	0.052	0.314	8.089	0.000	0.297	3.369
	Energy and fun	0.258	0.047	0.169	5.533	0.000	0.480	2.083
	Overload	0.494	0.074	0.225	6.716	0.000	0.397	2.521
	Fear and anxiety	1.278	0.148	0.325	8.639	0.000	0.315	3.172

^a^ Dependent variable: Tension, irritability, and fatigue.

## Data Availability

The data can be found at figshare.com under the following DOI: https://doi.org/10.6084/m9.figshare.27041626.
